# Techniques for aseptic dressing and procedures

**Published:** 2015

**Authors:** Dianne Pickering, Janet Marsden

**Affiliations:** Nurse Advisor (retired): *Community Eye Health Journal,* London, UK. dianne_logan@hotmail.com; Nurse Advisor: *Community Eye Health Journal,* London, UK. Email: J.Marsden@mmu.ac.uk

**Figure:**
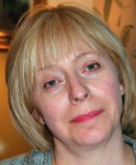
Dianne Pickering

**Figure:**
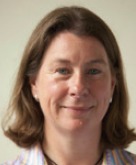
Janet Marsden

When applying or changing dressings, an aseptic technique is used in order to avoid introducing infections into a wound. Even if a wound is already infected, an aseptic technique should be used as it is important that no further infection is introduced. This technique should be used when the patient has a surgical or non-surgical wound in or around the eye.

## What you will need

- A clear available work space, such as a stainless steel trolley. The space must be big enough for the dressing pack to be opened on
- A sterile dressing/procedure pack
- Access to hand washing sink or alcohol hand wash
- Non-sterile gloves to remove old dressing
- Apron
- Appropriate dressings
- Appropriate solution for cleaning the wound, if needed.

## Preparation

- Introduce yourself to the patient and explain what you are doing and why. If possible, provide privacy.
- Position the patient comfortably and make sure the surrounding area is clean and tidy before you start.
- Check the patient's care notes to update yourself on any changes in the patient's condition and to make sure the dressing is due to be changed.
- Wash your hands and put on an apron.
- Clean the trolley using soap and water, or disinfectant, and a cloth. Start at the top of the trolley and work down to the bottom legs of the trolley using single strokes with your damp cloth.
- Place the sterile dressing/procedure pack on the top of the trolley.
- Open the sterile dressing pack on top of the trolley. Open the sterile field using the corners of the paper.
- Open any other sterile items needed onto the sterile field without touching them.

## Removing an old dressing

- Wash your hands and put on non-sterile gloves (to protect yourself) before removing an old dressing. Dispose of this dressing in a separate dirty clinical waste bag.

**Figure:**
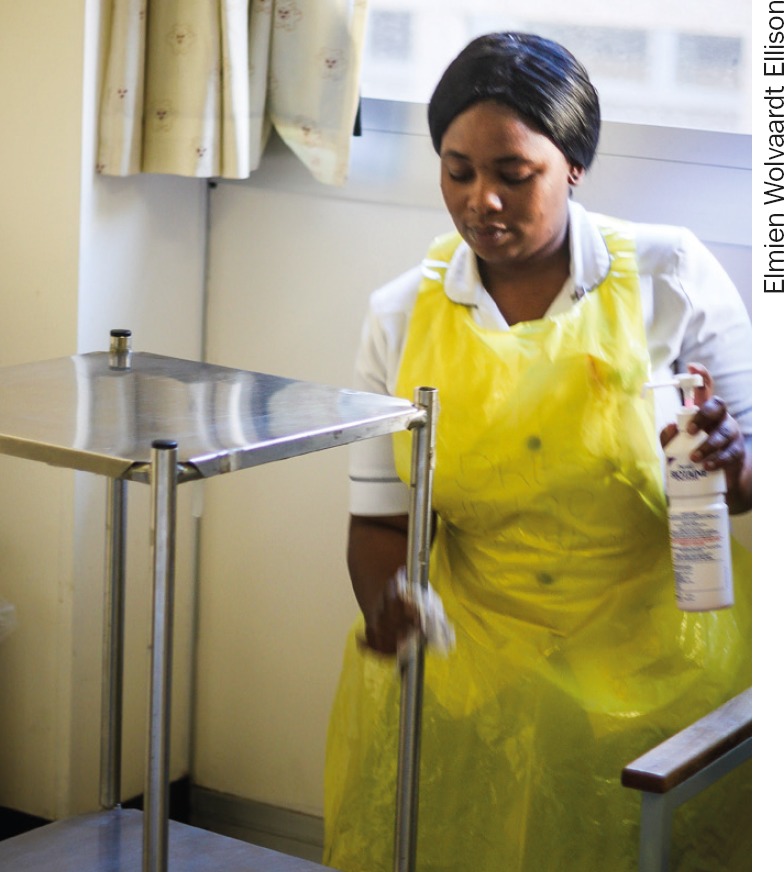
Start at the top and clean the trolley using single downward strokes
- Complete a wound assessment. This includes a visual check and comparing and evaluating the smell, amount of blood or ooze (excretions) and their colour, and the size of the wound.
- If the site has not improved as expected, then the treating physician or senior charge nurse must be informed so they too can evaluate it and consider changing the care plan.

**‘If the site has not improved as expected, inform the treating physician or senior nurse.’**

## Cleaning and dressing the wound

- Make sure that you have selected the correct dressing type and materials to provide full and appropriate coverage of the type, size and location of the wound as per the care plan or the physician or senior charge nurse's recommendations.
- Wash your hands and put on sterile gloves. If the gloves become desterilised, remove them, re-wash your hands and put on new sterile gloves. This is best practice, but where resources are not available, safe modifications to this process can be made, for example by using non-sterile gloves to protect the nurse while removing the dressing and then washing the hands with gloves on and using alcohol gel on the gloves to make them clean enough to clean the wound and redo the dressing. This then protects both the nurse and the patient.
- Start from the dirty area and then move out to the clean area. Be very careful when doing this as the tissue or skin may be tender and there may also be sutures in place. Clean the area without causing further damage or distress to the patient.

**Figure:**
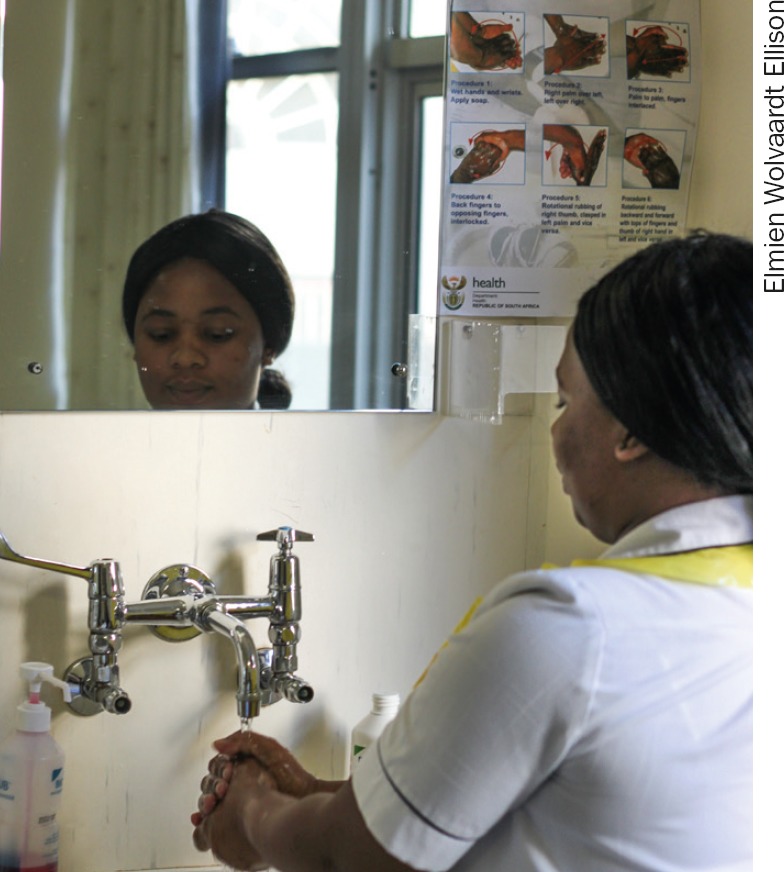
If your gloves become desterilised, wash your hands and put on fresh gloves
- Make sure you do not re-introduce dirt or ooze by ensuring that cleaning materials (i.e. gauze, cotton balls) are not over-used. Change them regularly (use once only if possible) and never re-introduce them to a clean area once they have been contaminated.
- Make sure that you have selected the correct dressing type and materials needed to provide full and appropriate coverage for the type, size and location of the wound, according to the care plan or the physician's or senior charge nurse's recommendations.
- Dress the wound as per instructions.
- **Note:** Ensure that the materials and dressing pack are only used for one eye at a time to prevent cross-contamination. If, for some reason, another part of the face or the other eye also needs a dressing change, then open another pack and start on the other side with clean hands and gloves.

## After the procedure

- Fold up the dressing/procedure pack and place all contaminated material in a bag designated for clinical waste, making sure all sharps are removed and disposed of in a sharps container.
- Remove gloves and place in waste bag.
- Wash your hands.
- Clean the trolley with soap and water or disinfectant solution as before.
- Record (document) on the patient's chart your wound assessment, the dressing change and the care you have given.
- Provide the patient with some dressing management education and answer any questions before you go.
- Report any changes to a senior nurse or doctor.

